# Efficacy of atopic dermatitis alleviation through transcutaneous auricular vagus nerve stimulation in 2,4-dinitrochlorobenzene (DNCB)-induced mouse model

**DOI:** 10.1080/07853890.2026.2680845

**Published:** 2026-06-09

**Authors:** Chan Mi Lee, Jae Hee Shin, Jae-Jun Song

**Affiliations:** ^a^ Department of Otorhinolaryngology-Head and Neck Surgery, Korea University Guro Hospital, Seoul, South Korea; ^b^ Institute for Health Care Convergence Center, Korea University Guro Hospital, Seoul, South Korea; ^c^ Neurive Institute, Neurive Co., Ltd, Seoul, South Korea

**Keywords:** Atopic dermatitis, transcutaneous auricular vagus nerve stimulation, α7 nicotinic acetylcholine receptor (α7nAChR), NF-κB signaling, DNCB-induced mouse model

## Abstract

**Background:**

Atopic dermatitis (AD) commonly begins in early childhood, and non-pharmacological treatment options remain limited. Vagal cholinergic signaling can modulate immune responses, suggesting a potential role for neuromodulation in inflammatory skin disease. This study investigated whether transcutaneous auricular vagus nerve stimulation (taVNS) alleviates AD-like skin inflammation and whether acetylcholine receptor signaling contributes to its effects.

**Methods:**

AD-like dermatitis was induced in BALB/c mice using 2,4-dinitrochlorobenzene (DNCB). taVNS (0.2 mA, 15 Hz for 5 min) was applied every 2–3 days for 1 or 2 weeks. Histopathology changes and inflammatory marker expression were assessed in lesional skin. To explore the underlying mechanism, acetylcholine receptor antagonism was used.

**Results:**

taVNS improved histological features and reduced inflammatory markers compared with DNCB alone. These effects were partially attenuated by acetylcholine receptor antagonism, suggesting involvement of cholinergic signaling, including α7 nicotinic acetylcholine receptors.

**Conclusion:**

taVNS may be a promising non-invasive adjunctive approach for AD. Its anti-inflammatory effects appear to be associated, at least in part, with cholinergic signaling pathways, warranting further mechanistic and translational studies.

## 1. Introduction

Atopic dermatitis (AD) is among the most prevalent skin conditions affecting infants and children [1]. Approximately 45% of children develop AD within the first six months of life, and about 85% of cases manifest before the age of 5 years [2–4]. AD is characterized by chronic, relapsing eczematous lesions and intense pruritus, driven by complex interactions between genetic susceptibility, skin barrier dysfunction, and immune dysregulation. Contact with inflammatory substances such as 2,4-dinitrochlorobenzene (DNCB) and other environmental antigens leads to their uptake by epidermal Langerhans cells, which migrate to regional lymph nodes and promote T helper type 2 (Th2)-skewed responses [5,6]. Th2 cells and their key cytokines, including interleukin (IL)-4, IL-13, IL-5 and IL-9, orchestrate downstream inflammatory cascades through their receptors on keratinocytes and various immune cells [7–10]. This type 2-dominant milieu enhances expression of chemokines such as thymus and activation-regulated chemokine (TARC), recruits Th2 cells to the skin, and activates mast cells, eosinophils, and basophils [11,12]. The resulting itch-scratch cycle leads to lichenification, pigmentary changes, and significant impairment in quality of life for affected individuals [13]. Despite the availability of topical and systemic therapies, including biologic and Janus kinase inhibitors, many patients experience incomplete disease control or treatment-related adverse effects [14,15]. Thus, durable control of chronic AD remains challenging, highlighting the need for novel therapeutic strategies.

Vagus nerve stimulation (VNS) has emerged as a neuromodulatory approach with documented anti-inflammatory effects in preclinical and clinical studies [16]. It has been explored as a novel therapeutic modality in inflammatory bowel diseases and investigated in conditions such as epilepsy, depression, postoperative ileus, and rheumatoid arthritis [17,18]. Conventional VNS typically requires surgical implantation of a pulse generator in the chest with leads placed around the cervical vagus nerve, which entails procedure-related risks and costs, as well as periodic battery replacement [19]. In addition to invasive VNS, non-invasive auricular stimulation has also been reported to modulate inflammatory responses in both experimental and clinical settings. These findings support the idea that auricular vagal stimulation can influence peripheral immune regulation and provide a rationale for testing taVNS in inflammatory skin disease [16,20,21]. To overcome these limitations, non-invasive transcutaneous VNS (tVNS) devices have been developed, delivering stimulation through the skin over vagal branches. Building on this concept, we developed an auricular VNS device that targets the ear and evaluated its efficacy in a DNCB-induced mouse model of AD.

AD is also closely linked to neurogenic, psychological, and social factors [22]. Psychological stress and autonomic imbalance can modulate allergic inflammation by altering sympathetic and parasympathetic activity, affecting eosinophil counts, immunoglobulin E (IgE) levels, and cytokine profiles such as IL-4 [23]. Given the anti-inflammatory effects associated with efferent vagal activity, reductions in vagal tone and dysregulated autonomic control may contribute to persistent inflammation in AD. Conversely, enhanced parasympathetic activity has been reported to exert anti-inflammatory effects and to ameliorate in inflammatory conditions. In this context, recent studies have investigated VNS as a mean to reduce inflammation in diseases such as rheumatoid arthritis and inflammatory bowel disease [24,25].

The anti-inflammatory actions of VNS are thought to be mediated in part by the cholinergic anti-inflammatory pathway, in which vagal efferent activity promotes acetylcholine release that signals through nicotinic acetylcholine receptors, particularly the α7 nicotinic acetylcholine receptor (α7nAChR), on immune cells [26,27]. Based on these considerations, we hypothesized that transcutaneous auricular vagus nerve stimulation (taVNS) could alleviate DNCB-induced AD-like skin inflammation by modulating cholinergic signaling *via* α7nAChR. In the present study, we investigated the effects of taVNS on histopathological changes and inflammatory markers in a mouse model of AD and evaluated the contribution of α7nAChR using pharmacologic antagonism.

## 2. Materials and methods

### 2.1. DNCB-induced atopy mouse model

All animal experiments were conducted with approval from the Institutional Animal Care and Use Committee (Korea-2023-0098) of Korea University College of Medicine. Male 7-week-old BALB/c mice (Orient Bio, Seongnam, Korea) were housed under standard conditions for 1 week before the induction of AD. Male mice were used to reduce biological variability and to maintain consistency across experimental cohorts in this exploratory study. Hair on the back skin and ears was removed 1 day prior to sensitization.

**Figure 1.**
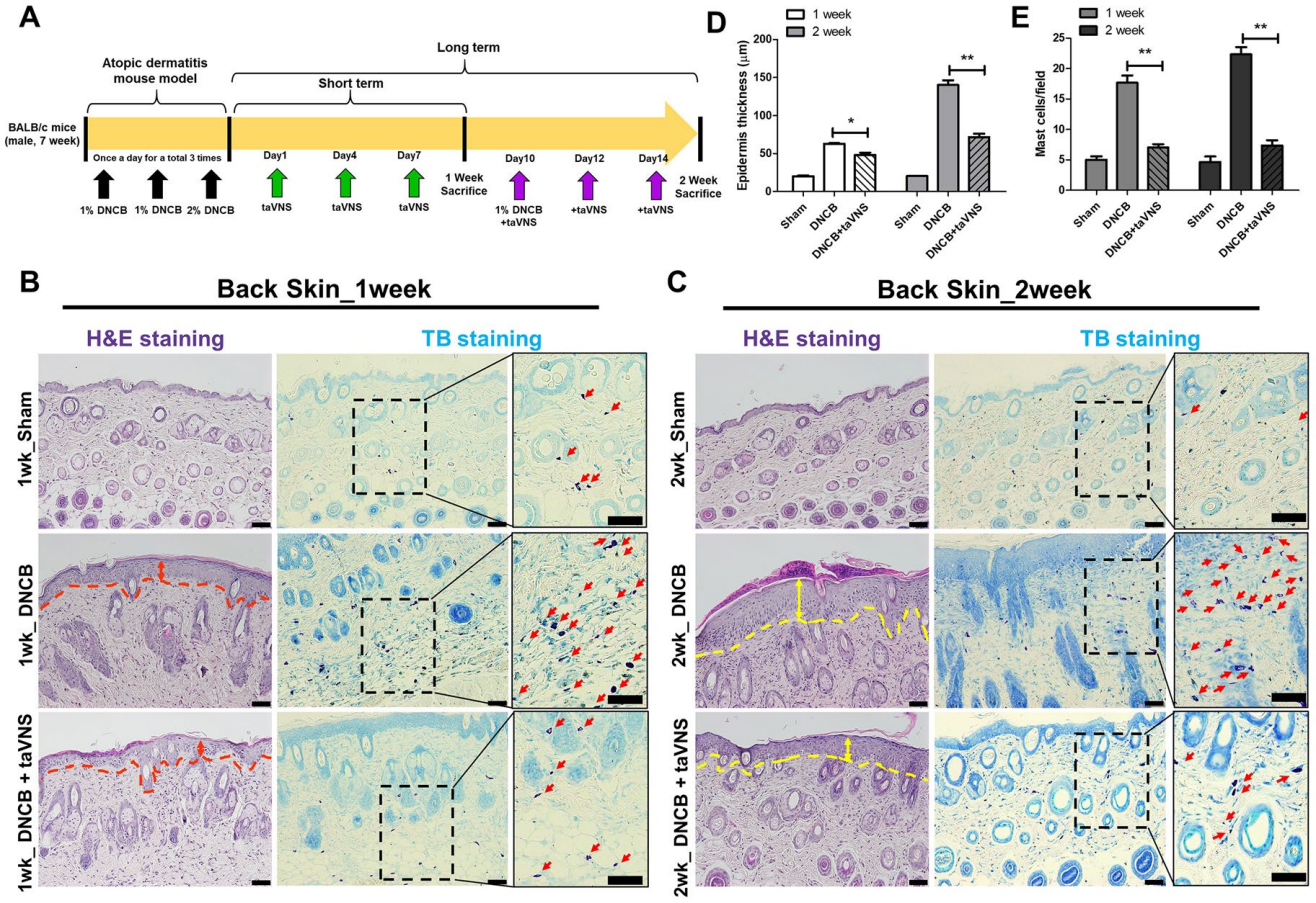
Effects of transcutaneous auricular vagus nerve stimulation (taVNS) on back skin in a DNCB-induced AD mouse model. (A) Schematic diagram of the experimental design. (B) Representative hematoxylin and eosin (H&E) and toluidine blue (TB)-stained sections of back skin after 1 week. Red solid lines indicate epidermal thickness, and red arrows indicate mast cells in TB-stained sections. (C) Representative H&E and TB-stained sections of back skin after 2 weeks. Yellow solid lines indicate epidermal thickness, and red arrows indicate mast cells. The taVNS group exhibited a reduction in mast cell-stained areas compared with the DNCB group. (D and E) Quantification of epidermal thickness and mast cell counts using ImageJ (*p < 0.05, **p < 0.01, and ***p < 0.001, *n* = 5/group). Scale bars, 50 μm.

To prepare a 2% 2,4-dinitrochlorobenzene (DNCB) solution, 400 mg of DNCB powder (Sigma, MO, USA) was dissolved in 20 mL of acetone/olive oil (4:1, v/v; acetone, JUNSEI, Japan) according to the manufacturer’s instructions [26]. On the back skin, a total of three applications were performed over 3 consecutive days: 100 µl of 1% DNCB was applied on day 1, followed by 120 µl of 2% DNCB on days 2 and 3. On the ear skin, 20 µl of 1% DNCB was applied on day 1, and 20 µl of 2% DNCB was applied on days 2 and 3 (Figure 1A). To ensure an unbiased distribution, mice were assigned to groups using a simple randomization method (*n* = 5/group). This process was conducted independently of the investigator’s subjective judgment, and the sequence was determined prior to the start of the induction to maintain objectivity. A total of 50 mice were used in this study, and the sample size was determined based on expected biological differences from previous studies to ensure statistical significance while minimizing animal use. To minimize potential distress, anesthesia was maintained using isoflurane vaporized in oxygen during all experimental procedures. The anesthesia was induced in an induction chamber with isoflurane delivered in > 90% medical oxygen at a flow rate of 2 L/min. Once the mice lost the righting reflex and failed to respond to a toe pinch, anesthesia was maintained with 2% isoflurane in > 90% oxygen *via* a nose cone at a flow rate of 2 L/min. At the end of the study, mice were euthanized *via* CO_2_ inhalation for tissue harvesting. All mice that completed the experimental protocol were included in the analysis; no prespecified exclusion criteria were applied, and no mice were excluded.

### 2.2. Transcutaneous auricular vagus nerve stimulation (taVNS)

Following completion of the DNCB-induced AD protocol, transcutaneous auricular vagus nerve stimulation (taVNS) was delivered using a taVNS device (Neurive Inc., Seoul, Korea). Stimulation was applied at 0.2 mA 15 Hz for 5 min under isoflurane respiratory anesthesia at intervals of 2-3 days [21]. For each session, mice in all experimental groups received the same isoflurane anesthesia for an identical duration. In the Sham group, the electrodes were placed on the ear for 5 min without delivering any electrical current. No direct physiological or molecular marker of vagal engagement was assessed in the present study.

### 2.3. α7 Nicotinic acetylcholine receptor (α7nAChR) antagonist

For experiments evaluating the involvement of α7 nicotinic acetylcholine receptors (α7nAChR), the DNCB-induced AD model was established as described in Section 2.1. On the day following completion of the 3-day DNCB induction, mice received methyllycaconitine citrate (MLA; Tocris, Bristol, UK), an α7nAChR antagonist, at a dose of 0.25 mg/kg by intraperitoneal injection and 0.25 mg/kg as a topical application to the skin, 1 h before taVNS. The dose and timing of MLA were selected based on prior studies reporting effective *in vivo* antagonism of α7nAChR [20,21]. This experimental protocol (MLA administration followed by taVNS) was performed every other day for a total of three sessions, after which back and ear skin tissues were collected for subsequent analyses (Figure 4A).

### 2.4. Western blot

Back and ear skin tissues were homogenized in RIPA buffer (Sigma, R0278) containing protease inhibitor (1:100, GenDEPOT USA) using a tissue homogenizer. Homogenates were centrifuged at 2000×*g* for 10 min, and the supernatants were collected. Protein concentrations were determined with a bicinchoninic acid (BCA) protein assay kit (Thermo Scientific, Rockford, USA) according to the manufacturer’s instructions [28]. Samples were mixed with 5× SDS-PAGE sample buffer (Biosesang, Yongin, Korea) and heated at 95 °C for 5 min before electrophoresis. Proteins were separated by SDS-PAGE and transferred to membranes, which were then incubated with primary antibodies against TNF-α (1:500, Santa Cruz), MPO (1:1000, Abcam) and β-actin (1:1000, Cell Signaling Technology) overnight at 4 °C. After three washes with TBS-T (Biosesang), membranes were incubated with HRP-conjugated secondary antibodies (anti-mouse IgG-HRP, 1:3000; anti-rabbit IgG-HRP, 1:3000; Cell Signaling Technology) for 2 h at room temperature. Protein bands were visualized using an enhanced chemiluminescent substrate (Thermo Scientific) and detected with a fluorescent imaging system (Fusion-Solo.6S, Vilber Lourmat, France).

### 2.5. Real-time quantitative PCR

Back and ear skin tissues were cut into approximately 0.5 cm pieces and homogenized in 1 mL of TRIzol reagent (Invitrogen, CA, USA). Phase separation was performed by adding chloroform (Sigma), followed by centrifugation; the aqueous phase was collected and mixed with isopropanol (Sigma) to precipitate RNA. The RNA pellet was washed with 100% and 70% ethanol, air-dried, and dissolved in diethyl pyrocarbonate (DEPC)-treated water (SGbio, Seoul, Korea). Total RNA concentration and purity were measured using a NanoDrop spectrophotometer (Thermo Scientific). cDNA was synthesized from 1 µg of total RNA using the PrimeScript^TM^ 1st strand cDNA synthesis kit (Takara Bio, Kusatsu, Shiga, Japan) according to the manufacturer’s instructions [29]. For quantitative real-time PCR, 50 ng of cDNA (1 µL) was mixed with 10 µL of Power SYBR Green PCR Master Mix (Life Technologies, Warrington, UK), 0.5 µL each of forward and reverse primers (10 pmol), and nuclease-free water to a final volume of 20 µL. Primer sequences are listed in Table 1. Reactions were run on a QuantStudio 6 Flex system with 40 cycles of amplification, including an annealing step at 60 °C for 1 min. All samples were analyzed in triplicate, and mRNA expression levels were normalized to GAPDH.

**Table 1.** Inflammation marker primer sequences used for real-time quantitative PCR.

Primer	Direction	sequence (5′–3′)
*TNF-α*	F	CGT CAG CCG ATT TGC TAT CA
	R	CGG ACT CCG CAA AGT CTA AG
*IL-1β*	F	TCG CAG CAG CAC ATC AAC AAG
	R	CAT GTC CTC ATC CTG GAA G
*IL-4*	F	TCA ACC CCC AGC TAG TTG TC
	R	TGT TCT TCG TTG CTG TGA GG
IL-13	F	CAGCATGGTATGGAGTGTGG
	R	GTGGGCTACTTCGATTTTGG
*GAPDH*	F	ACC CAG AAG ACT GTG GAT GG
	R	TCA GCT CTG GGA TGA CCT TG

### 2.6. Hematoxylin and eosin (H&E) staining

Back and ear skin tissues were fixed in 4% paraformaldehyde (Biosesang) at 4 °C for 1 week and then processed for paraffin embedding and sectioning by the Department of Pathology, Korea University. Paraffin sections were deparaffinized in xylene, rehydrated through graded ethanol, and rinsed in distilled water. Slides were stained with Mayer’s hematoxylin (ScyTek, Logan, USA), differentiated in 1% hydrochloric acid in 70% ethanol, and blued in 0.2% ammonia water. After counterstaining with eosin Y alcoholic solution (BBC Biochemical, Mount Vernon, USA), sections were dehydrated through graded ethanol, cleared in xylene, and mounted with a permanent mounting medium (Sigma). Epidermal thickness was quantified using ImageJ software.

### 2.7. Toluidine blue (TB) staining

For mast cell staining, paraffin sections were deparaffinized, rehydrated, and rinsed in distilled water. A toluidine blue (TB) stock solution was prepared with 1% toluidine blue O in 70% ethanol, a working solution was made by mixing 5 mL of TB stock with 45 mL of 1% sodium chloride solution, and the pH was adjusted to 2.3 with hydrochloric acid. Sections were incubated in the TB working solution for 3 min, rinsed in distilled water, dehydrated through graded ethanol, cleared in xylene, and mounted. Mast cells were visualized under a light microscope (Olympus, Tokyo, Japan), and the number of mast cells was measured in at least three randomly selected fields per section at 20× magnification.

### 2.8. Statistical analysis

All data results were expressed as mean ± standard deviation. Statistical analyses were performed using GraphPad Prism software (version 5.0; GraphPad Software, San Diego, CA, USA). Group comparisons were evaluated using one-way analysis of variance (ANOVA) followed by the Tukey post-hoc test. Statistical significance was indicated as *p < 0.05, **p < 0.01, and ***p < 0.001. All histological, molecular, and quantitative analyses were performed by investigators who were blinded to the group assignments.

## 3. Results

### 3.1. taVNS alleviated AD on the back skin in a DNCB-induced mouse model

To establish the AD mouse model, mice were sensitized with 1%–2% DNCB for 3 days. Short-term (1 week) and long-term (2 weeks) efficacy of taVNS was compared, with the short-term group receiving three sessions of taVNS over a week, and the long-term group receiving six sessions over two weeks before sacrificing the mice to retrieve back skin tissues (Figure 1A). In the 1-week, histological tissue staining of back tissues confirmed a visually observed increase in epidermal thickness in the DNCB group (62.89 µm ± 0.93) compared to the sham group (20.03 µm ± 1.44). The taVNS group (48.25 µm ± 4.28) exhibited a decrease in epidermal thickness compared to the DNCB group, suggesting that taVNS may contribute to a reduction in epidermal thickness. TB staining was conducted to visualize Mast cells, which play a crucial role in immune and inflammatory responses, particularly in AD. Mast cell distribution and quantity were assessed to understand their role in AD. Mast cells were counted in three different areas, with the DNCB group (22.33 ± 1.25) showing an increase compared to the sham group (3 ± 0.82), and the taVNS group (13.67 ± 1.25) also exhibiting a decrease (Figure 1B). In the 2 weeks, epidermal thickness in back tissues decreased in the taVNS group (71.75 µm ± 6.17) compared to the DNCB group (140.04 µm ± 9.00). The number of Mast cells in the taVNS group (12 ± 0.82) decreased compared to the DNCB group (22.33 ± 3.68) (Figure 1C). Quantitative analysis of epidermal thickness and Mast cell count in both weeks revealed a significant decrease in the taVNS group compared to the DNCB group (Figure 1D and E).

### 3.2. taVNS alleviated AD in the ear skin of a DNCB-induced mouse model

**Figure 2.**
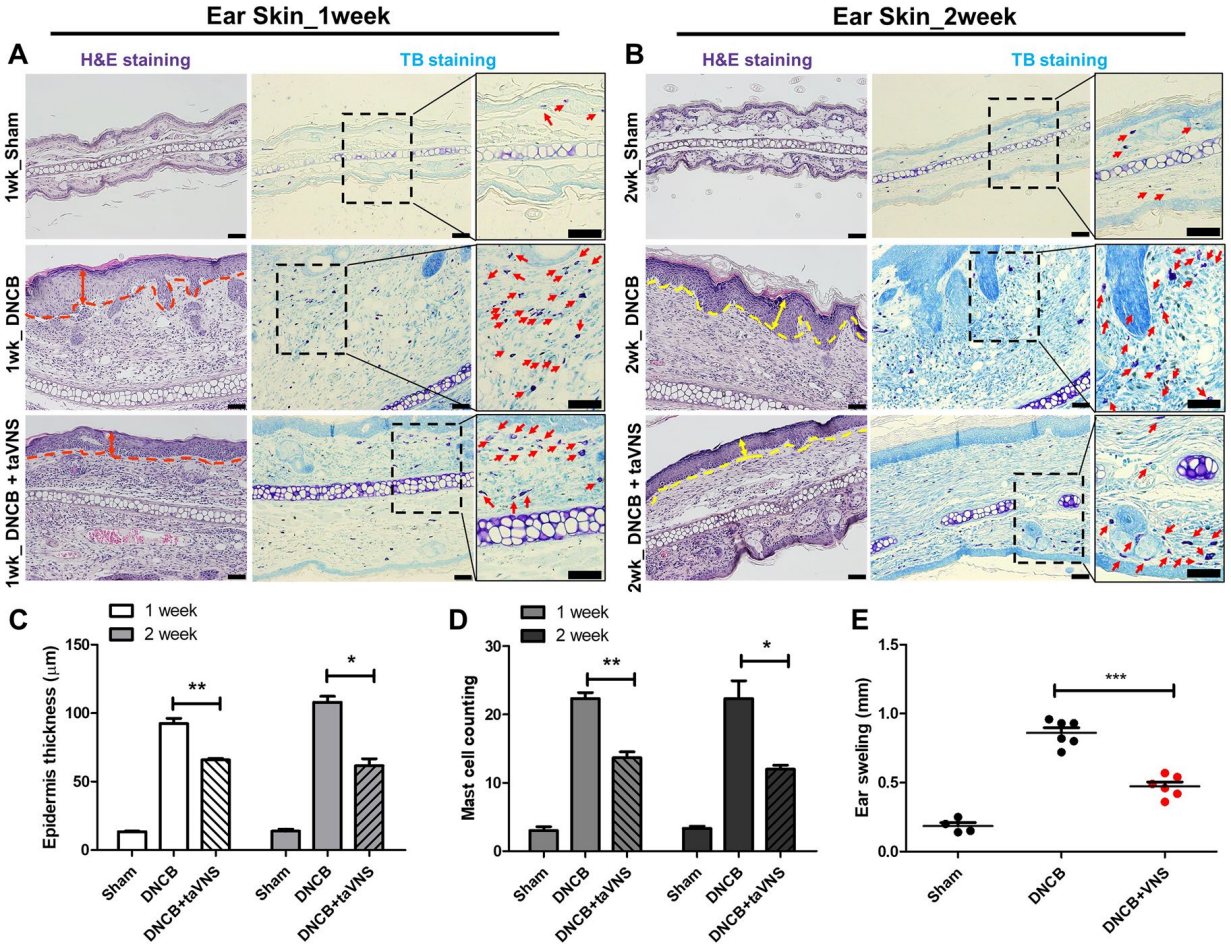
Effects of taVNS on ear skin in a DNCB-induced AD mouse model. (A and B) Representative H&E and TB-stained sections of ear skin after 1 and 2 weeks. Solid lines indicate epidermal thickness, and red arrows indicate mast cells in TB-stained sections. (C) Quantification of epidermal thickness using ImageJ showing a significant reduction in the taVNS group compared with the DNCB group. (D) Quantification of mast cell counts using ImageJ showing a significant decrease in the taVNS group compared with the DNCB group. (E) Ear swelling measured by calipers after sacrifice showing a significant reduction in the taVNS group compared with the DNCB group. *p < 0.05, **p < 0.01, and ***p < 0.001., *n* = 5/group in Epidermis thickness and Mast cell counting, *n* = 4 ∼ 6/group in Ear swelling., Scale bars, 50 μm.

In this study, the therapeutic efficacy of taVNS was investigated in mitigating AD within a DNCB-induced mouse model. Histological assessments, utilizing H&E staining, and TB staining, were conducted on ear tissues during the first week to visually depict epidermal thickness. The ear’s epidermal thickness in the DNCB group (92.30 μm ± 5.47) increased compared to the sham group (13.40 μm ± 0.75). the taVNS group (65.84 μm ± 1.48) exhibited a significant reduction compared to the DNCB group. Mast cell counts in the ear increased in the DNCB group (17.67 ± 1.70) compared to the sham group (5.00 ± 0.82). In contrast, the taVNS group (7.00 ± 0.82) demonstrated a significant decrease (Figure 2A). In the second week, epidermal thickness in the taVNS group (61.53 μm ± 7.33) significantly decreased compared to the DNCB group (107.95 μm ± 6.10). Mast cell counts in the taVNS group (7.33 ± 1.25) were significantly reduced compared to the DNCB group (22.33 ± 1.70) (Figure 2B). Quantitative analysis of epidermal thickness for both the first and second weeks, graphically represented, showed a significant reduction in the taVNS group compared to the DNCB group (Figure 2C). Mast cell counts, recorded in different regions, exhibited a significant decrease in the taVNS group compared to the DNCB group (Figure 2D). Additionally, ear swelling in the second week was measured using calipers, revealing a significant reduction in the taVNS group (0.48 mm ± 0.07) compared to the DNCB group (0.87 mm ± 0.08) (Figure 2E). We propose that taVNS contributes to a reduction in epidermal thickness in the ear, suggesting its potential as a therapeutic intervention for AD.

### 3.3. taVNS decreases inflammatory markers in skin tissues of DNCB-induced AD mouse model

**Figure 3.**
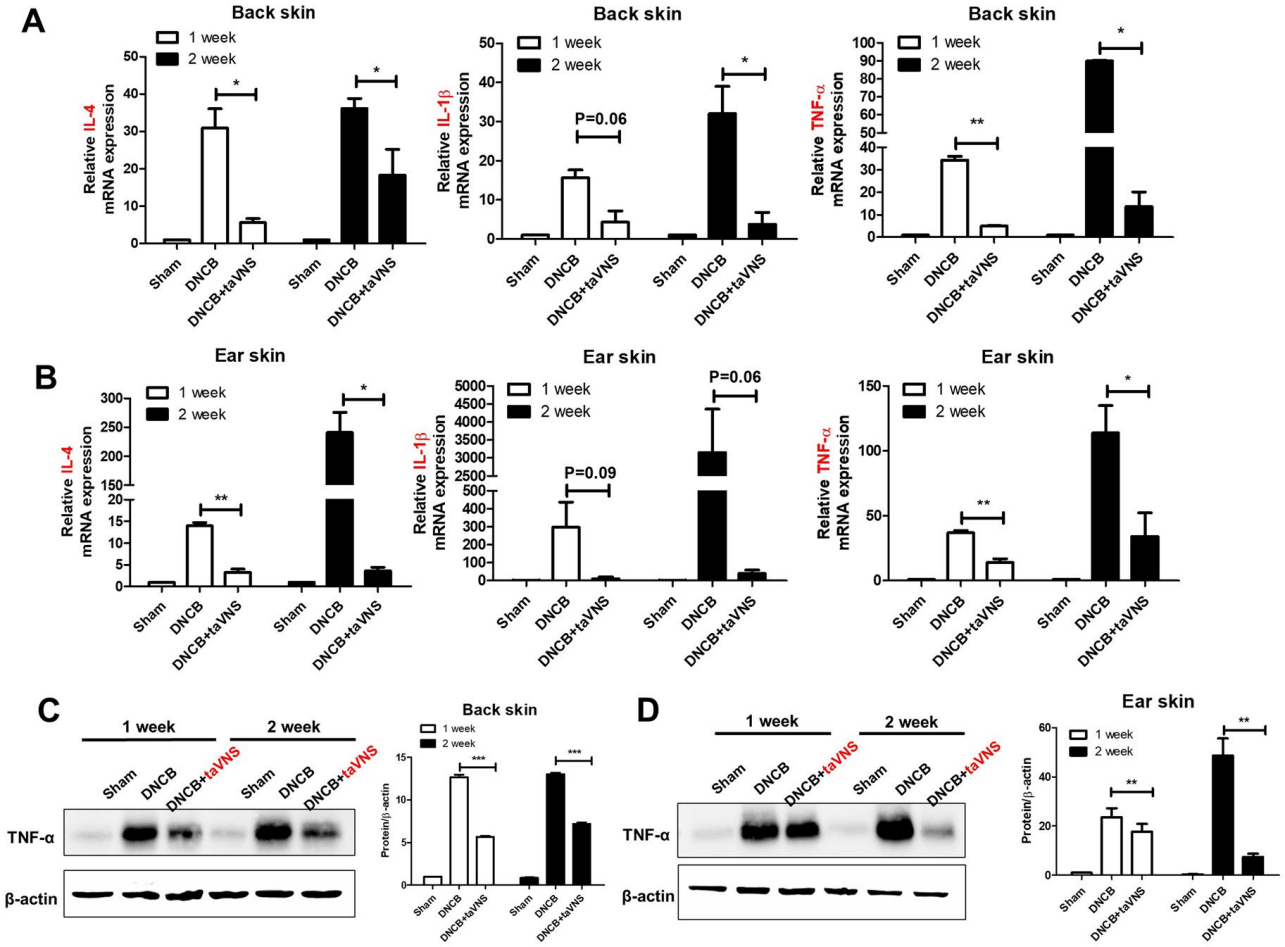
taVNS reduces inflammatory markers in a DNCB-induced AD mouse model. (A) mRNA expression levels of IL-4, IL-1β, and TNF-α in back skin after 1 and 2 weeks were analyzed by quantitative RT-PCR. The taVNS group showed significantly lower expression compared with the DNCB group. (B) mRNA expression levels of IL-4, IL-1β, and TNF-α in ear skin after 1 and 2 weeks were also reduced in the taVNS group compared with the DNCB group. (C and D) Protein expression of TNF-α and MPO in skin tissues was assessed by Western blot, demonstrating a significant decrease in the taVNS group compared with the DNCB group. *p < 0.05, **p < 0.01, and ***p < 0.001., *n* = 5/group.

taVNS demonstrated a reduction in inflammatory markers in the skin tissues of a DNCB-induced AD mouse model. The cytokine inflammatory markers regulating inflammation in back and ear tissues (IL-1β, TNF-α) were quantitatively measured for mRNA expression levels using qRT-PCR. All inflammatory markers showed a consistent decrease in the taVNS group compared to the DNCB group at both 1 and 2 weeks (Figure 3A). Additionally, mRNA expression levels of inflammatory markers in ear tissues (1 week and 2 weeks) were lower in the taVNS group compared to the DNCB group (Figure 3B). The protein expression levels of TNF-α were visualized using Western blotting. In both back and ear tissues (1 and 2 weeks), the taVNS group exhibited a significant reduction in TNF-α expression levels compared to the DNCB group, as depicted in quantitative graphs (Figure 3C and D). Assessing mRNA and protein expression levels of inflammatory cytokines provides insights into the potential generation of skin inflammatory proteins during DNCB induction and helps comprehend the inhibitory effects of taVNS on inflammatory markers.

### 3.4. The α7nAChR antagonist attenuates the anti-inflammatory effects of taVNS in a DNCB-induced AD mouse model

**Figure 4.**
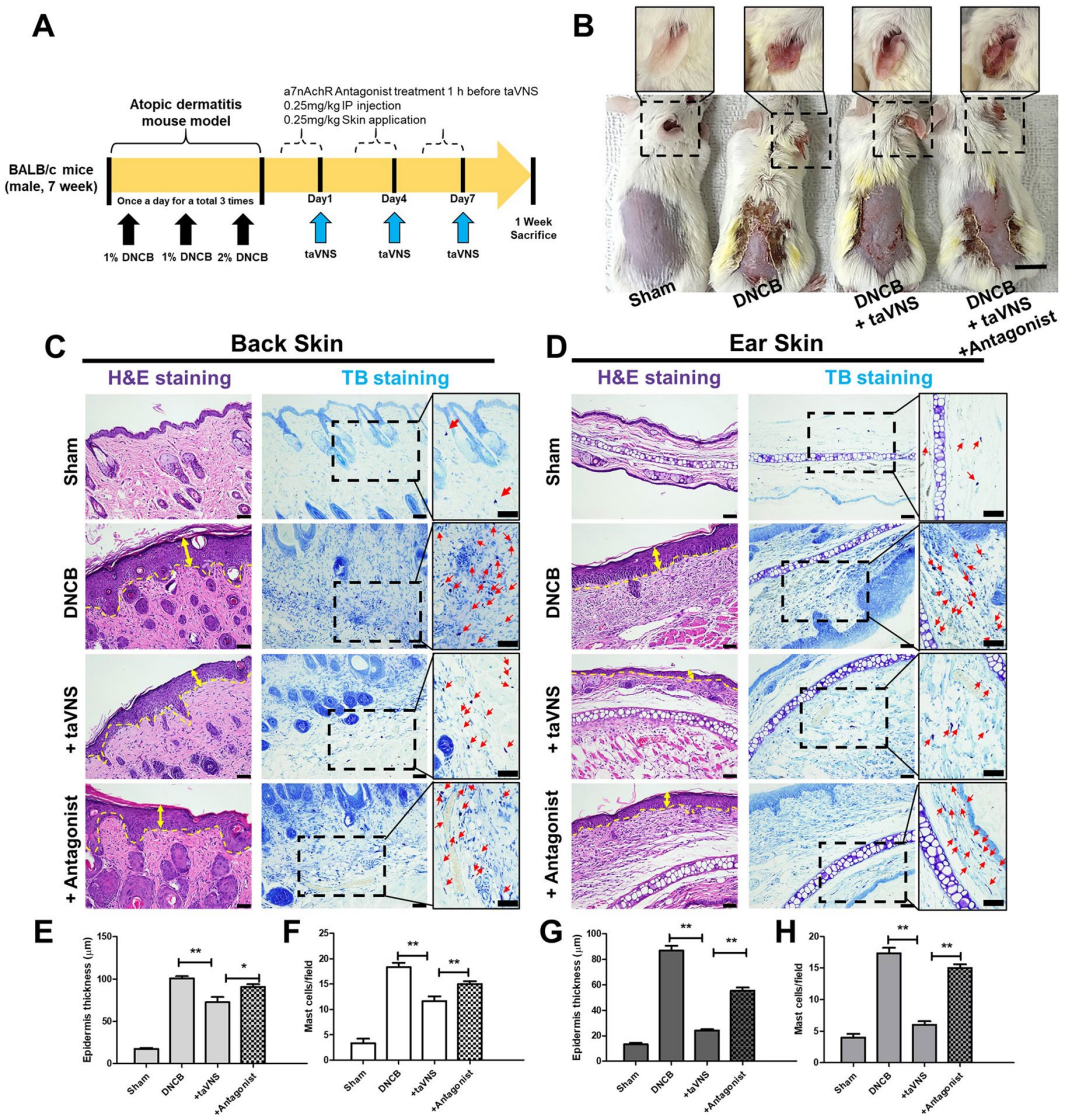
An acetylcholine receptor antagonist attenuates the anti-inflammatory effects of taVNS in a DNCB-induced AD mouse model. (A) Schematic representation of the experimental protocol and dosing schedule of the acetylcholine receptor antagonist. (B) Representative macroscopic images of each group at the end of the experiment. (C and D) Histological analysis of back and ear skin after 1 week. H&E staining shows epidermal thickness (yellow arrows), and TB staining highlights mast cells (red arrows). (E and G) Quantification of epidermal thickness in back and ear skin using ImageJ. The antagonist-treated group showed increased epidermal thickness compared with the taVNS group. (F and H) Mast cell counts in back and ear skin (mean of at least three randomly selected fields per section). The antagonist-treated group showed increased mast cell numbers compared with the taVNS group. *p < 0.05, **p < 0.01, and ***p < 0.001., *n* = 5/group., Scale bars, 50 μm.

In the DNCB-induced AD mouse model, the acetylcholine receptor antagonist exhibited a reduction in the anti-inflammatory effects observed in pathological analyses. Hypothesizing a correlation between the efficacy of taVNS and acetylcholine receptors, we conducted a pharmacological intervention using the α7nAChR antagonist. To compare the short-term (1 week) efficacy of the acetylcholine receptor antagonist, we established the AD mouse model and applied α7nAChR antagonist intraperitoneally and topically to the skin one hour before taVNS application. Mice were sacrificed, and back and ear tissues were recovered (Figure 4A). Representative mouse photos revealed the highest degree of skin damage in the DNCB group. In contrast, the taVNS group exhibited skin recovery. In contrast, the Antagonist group showed a broader area of damaged skin compared to the taVNS group (Figure 4B). Histological analysis with H&E staining of back and ear tissues measured the epidermal thickness of each group. The Antagonist group exhibited a significantly thicker epidermis compared to the taVNS group. TB staining was performed to quantify mast cell numbers, revealing an increased mast cell count in the Antagonist group compared to the taVNS group (Figure 4C and D). Epidermal thickness in back tissue was quantified as follows: Sham group (17.27 um ± 1.57), DNCB group (100.87 um ± 3.57), taVNS group (72.44 um ± 8.68), Antagonist group (90.74 um ± 4.41). The Antagonist group showed a significant increase in epidermal thickness compared to the taVNS group (Figure 4E). Mast cell numbers in back tissue were quantified as follows: Sham group (3.33 ± 1.25), DNCB group (18.33 ± 1.25), taVNS group (11.67 ± 1.25), Antagonist group (15.00 ± 0.82). The Antagonist group exhibited a significantly higher mast cell count compared to the taVNS group (Figure 4F). Epidermal thickness in ear tissue was quantified as follows: Sham group (13.37 um ± 1.55), DNCB group (86.97 um ± 5.25), taVNS group (24.30 um ± 1.31), Antagonist group (55.47 um ± 3.43). The Antagonist group showed a significant increase in epidermal thickness compared to the taVNS group (Figure 4G). Mast cell numbers in ear tissue were quantified as follows: Sham group (4.00 ± 0.82), DNCB group (17.33 ± 1.25), taVNS group (6.00 ± 0.82), Antagonist group (15.00 ± 0.82). The Antagonist group exhibited a significantly higher mast cell count compared to the taVNS group (Figure 4H). The increase in mast cells may be associated with inflammatory responses or excessive immune system activation. Therefore, the α7nAChR antagonist is proposed to inhibit the inflammation relief induced by taVNS.

### 3.5. Impact of acetylcholine receptor antagonist on inflammatory markers in taVNS-treated AD mouse model

**Figure 5.**
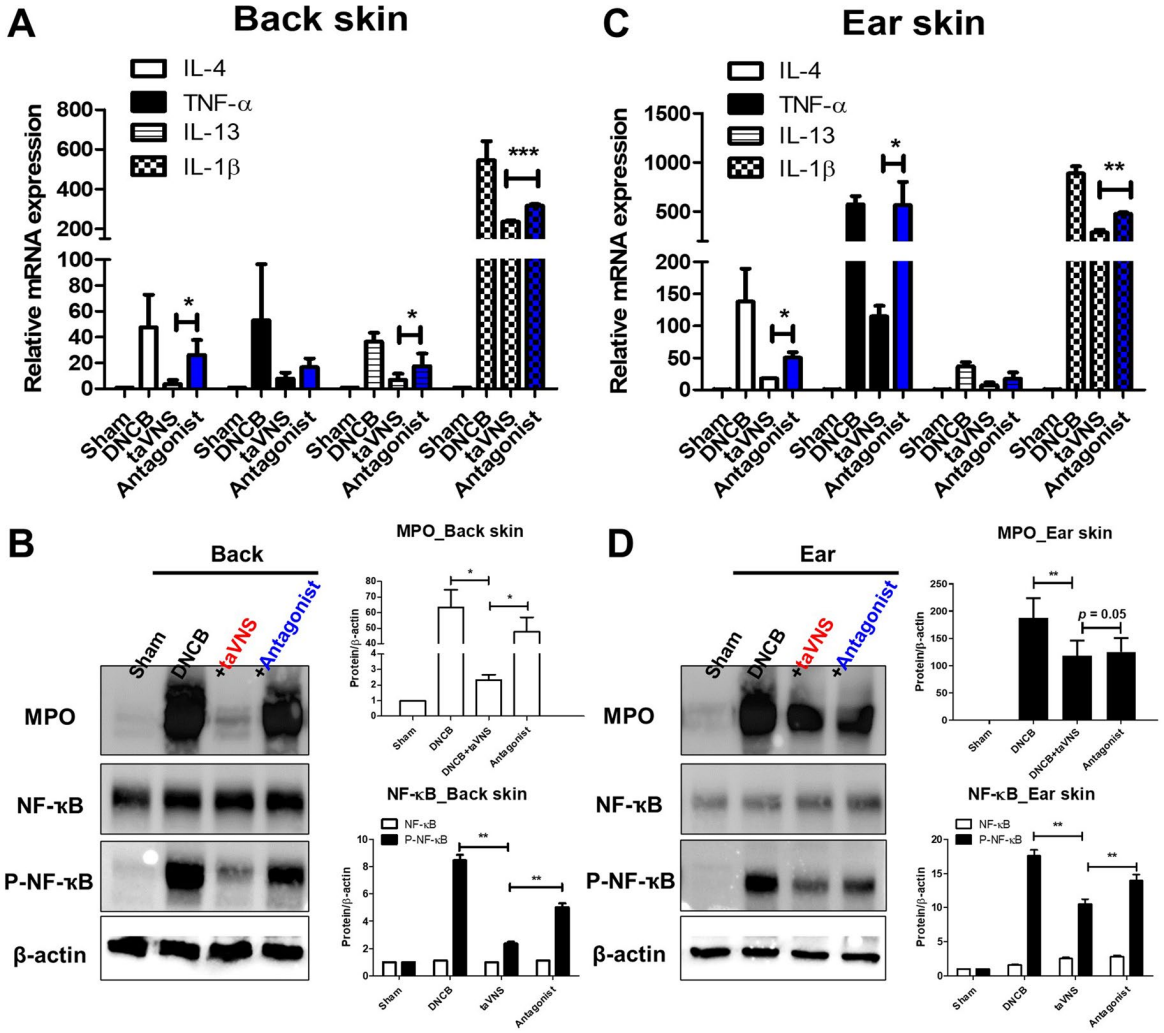
An acetylcholine receptor antagonist increases inflammatory markers in back and ear skin in a DNCB-induced AD mouse model. (A and C) mRNA expression levels of IL-4, IL-1β, and TNF-α in back and ear skin were higher in the acetylcholine receptor antagonist group than in the taVNS group. (B and D) Protein expression levels of MPO, NF-κB, and phosphorylated NF-κB (p-NF-κB) in back and ear skin were increased in the antagonist group compared with the taVNS group. Densitometric analysis was performed using ImageJ, and protein levels were normalized to β-actin. *p < 0.05, **p < 0.01, and ***p < 0.001., *n* = 5/group.

In the AD mouse model induced by DNCB, the acetylcholine receptor antagonist group they exhibited an increase in inflammatory markers in both back and ear skin regions following taVNS treatment. To measure the mRNA expression levels of inflammatory markers (IL-4, TNF-α, IL-13, IL-1β) in back and ear tissues, qRT-PCR was conducted. The mRNA expression levels of inflammatory markers in the Antagonist group showed a tendency to increase compared to the taVNS group (Figure 5A and C). Protein expression levels of MPO and NF-κB, crucial players in the inflammatory environment, were measured using western blotting. MPO, an enzyme primarily expressed in neutrophils, regulates inflammatory responses, and contributes to cellular damage through the generation of reactive oxygen species. MPO primarily acts in the early stages of inflammation, while NF-κB is involved in the regulation and maintenance of inflammation. The protein expression of MPO increased in both back and ear tissues in the DNCB group compared to the taVNS group, and the Antagonist group showed an increased expression compared to the taVNS group. In back and ear tissues, the protein expression of Phospho-NF-κB significantly increased in the DNCB group compared to the control group. The Antagonist group exhibited a significant increase compared to the DNCB group (Figure 5B and D). These findings suggest that the acetylcholine receptor antagonist may enhance inflammatory markers, emphasizing the intricate interplay between taVNS, acetylcholine receptors, and inflammatory responses in the context of AD.

## 4. Discussion

In this study, we demonstrated that transcutaneous auricular vagus nerve stimulation (taVNS) ameliorated cutaneous inflammation in a 2,4-dinitrochlorobenzene (DNCB)-induced mouse model of atopic dermatitis (AD). taVNS delivered repeatedly led to reductions in epidermal hyperplasia, mast cell density, and the expression of signature inflammatory mediators such as IL-4, IL-1β, TNF-α, MPO, and NF-κB pathway components in back and ear skin. Additionally, pharmacological inhibition of acetylcholine receptors diminished the anti-inflammatory benefits of taVNS, thereby implicating cholinergic signaling-especially *via* α7 nicotinic acetylcholine receptors (α7nAChR)-as a key mediator of its cutaneous effects. Mast cells are central effector cells in AD and contribute to pruritus, vasodilation, and recruitment of additional inflammatory cells [30]. In our model, DNCB challenge led to marked mast cells accumulation in the dermis, whereas taVNS significantly reduced mast cells number in both back and ear skin. Given that mast cells can activate the NF-κB pathway and promote the production of pro-inflammatory cytokines, the observed reduction in mast cells is consistent with the overall decrease in inflammatory readouts. Although we did not directly assess pruritus-related behavior, attenuation of mast cells infiltration by taVNS may be relevant for reducing itch and the itch-scratch cycle in AD.

Our results also suggest that taVNS affects inflammatory pathways related to NF-κB signaling. Under resting conditions, NF-κB is kept inactive in the cytoplasm by inhibitor of κB (IκB) proteins and becomes activated in response to stimuli such as contact allergens and microbial products [31]. In the DNCB model, an increase in NF-κB-related proteins and inflammatory cytokines is expected, reflecting enhanced transcription of pro-inflammatory genes. In our study, taVNS reduced NF-κB-associated protein levels and downstream cytokines, whereas treatment with an α7nAChR antagonist weakened these effects. These findings are consistent with the cholinergic anti-inflammatory pathway, in which activation of α7nAChRs on immune cells, including macrophages, inhibits NF-κB activation and cytokine release [32,33]. However, because we did not directly measure NF-κB nuclear translocation or IκB degradation, these mechanisms should be regarded as a possible explanation rather than a definitive proof. MPO is a marker of neutrophil-rich inflammation and can contribute to tissue damage by generating reactive oxygen species [34–36]. Increased MPO expression in DNCB-treated skin likely reflects the recruitment and activation of neutrophils in the inflamed tissue. In our study, taVNS reduced MPO protein levels, and this effect was reversed by acetylcholine receptor antagonism, suggesting that vagal modulation may reduce neutrophil-driven oxidative stress in AD-like lesions. Together with the changes in cytokines and mast cells, these findings indicate that taVNS has a broader immunomodulatory effect on multiple inflammatory cells population in the skin.

**Figure 6.**
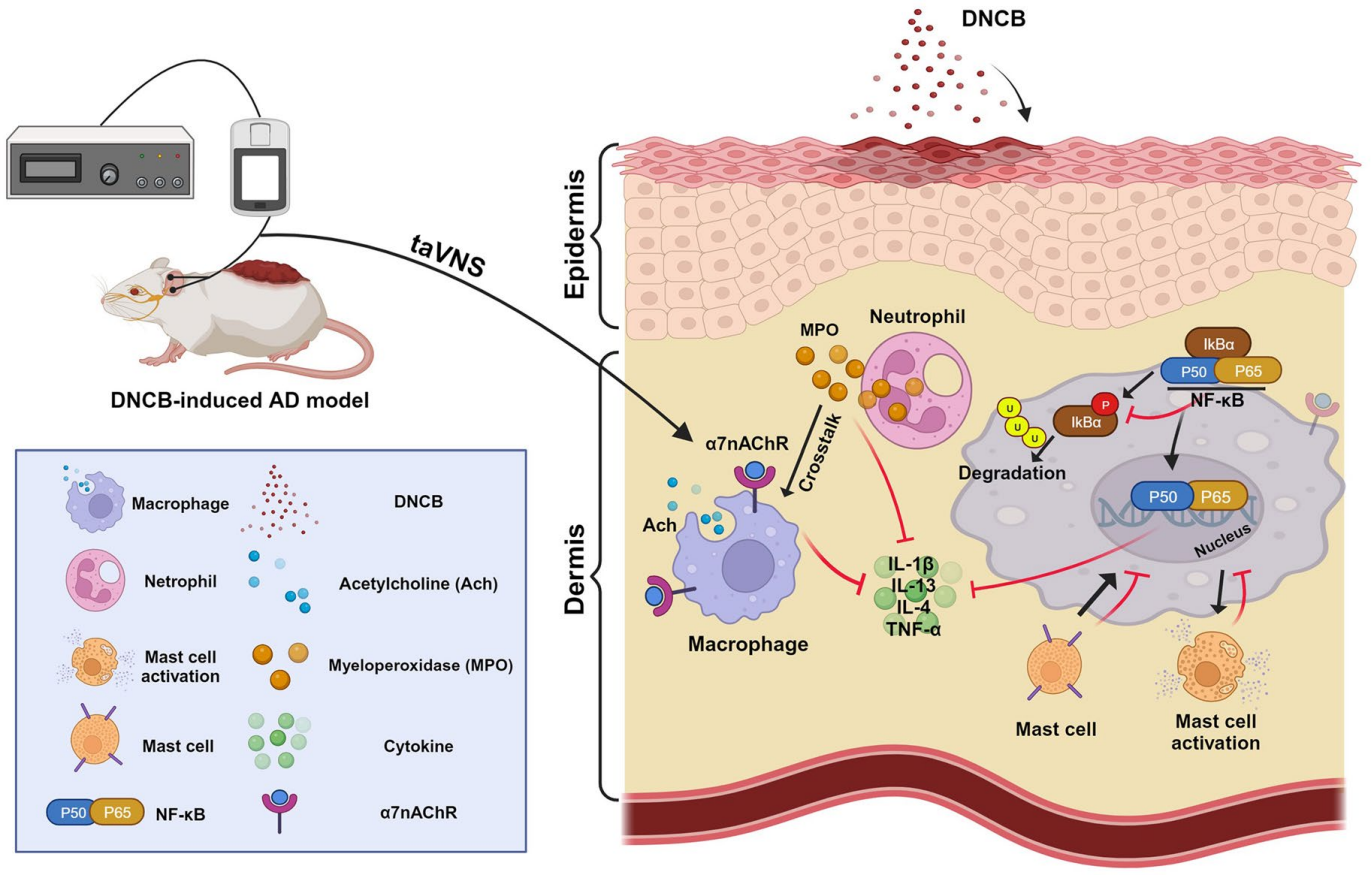
Schematic illustration of the proposed mechanism by which taVNS attenuates inflammation in a DNCB-induced AD mouse model.

The vagus nerve is an important link between the nervous and immune systems, and stimulation of vagal pathways can suppress both systemic and local inflammation through cholinergic mechanisms [16–18,24,37]. Additionally, sympathetic nerve dysfunction has been reported to exacerbate skin inflammation and pruritus in AD, indicating that autonomic imbalance contributes to AD pathophysiology [38]. In the skin, immune cells such as macrophages and other leukocytes in the dermis express α7nAChRs and can respond to acetylcholine released from autonomic nerve fibers [39,40]. In our study, the effects of taVNS were reduced by an acetylcholine receptor antagonist, indicating that the improvement seen in the DNCB model is at least partly dependent on acetylcholine receptor signaling. This is consistent with activation of α7nAChR-positive immune cells in the dermis, aligning with recent comprehensive review that highlight the bidirectional crosstalk between the peripheral nervous system and lymphoid tissues [41]. Although we did not directly measure acetylcholine levels or receptor binding, our pharmacologic results are in line with previous reports that implicate α7nAChRs in the anti-inflammatory effects of vagal stimulation in the skin. A schematic illustration of the proposed mechanism is shown in Figure 6.

From a translational perspective, taVNS is a non-pharmacological and non-invasive approach that may complement current treatments for AD. Existing treatments for AD are often limited by incomplete responses, adverse effects, and cost, even when using topical agents, systemic drugs, biologics, or Janus kinase inhibitors. Our results provide preclinical evidence that taVNS could serve as an adjunctive therapy and support further studies in more chronic and clinically relevant models. However, this study has several limitations. We used an acute DNCB-induced model, which does not fully reflect the chronic and relapsing nature of human AD. Notably, we did not assess itch-related behaviors (scratching), which are clinically important endpoints in AD; as this research was designed as a stepwise investigation primarily focused on the initial therapeutic efficacy of taVNS, the translational relevance of taVNS to pruritus should be interpreted with caution. Although we did not directly evaluate autonomic function or the direct activation of acetylcholine and α7nAChR, our pharmacological results using an antagonist suggest the potential involvement of the cholinergic anti-inflammatory pathway. Additionally, we did not directly assess vagal engagement using physiological measures such as heart rate variability or molecular markers of vagal activation. We also did not directly confirm receptor occupancy or downstream blockade following MLA treatment; therefore, the mechanistic interpretation is based on pharmacologic interference rather than direct receptor validation. Furthermore, this study was conducted exclusively in male mice to minimize experimental variability associated with hormonal cycle; however, as AD prevalence and immune responses can differ by sex, future investigations should include female cohorts to enhance the generalizability of these findings. While we did not include a group receiving the α7nAChR antagonist alone, our previous work demonstrated that this antagonist does not significantly alter baseline cytokine levels; nonetheless, the potential for non-specific effects should be considered in future studies. In addition, we examined only a limited set of inflammatory markers, which were selected as hallmark indicators to represent the core inflammatory response in this model. Future studies should test taVNS in chronic models, further optimize stimulation parameters, and include additional clinical and mechanistic readouts.

In summary, taVNS reduced DNCB-induced AD-like skin inflammation in mice, lowering epidermal thickness, mast cell numbers, and pro-inflammatory mediators in both back and ear skin. These anti-inflammatory effects were weakened by an acetylcholine receptor antagonist, suggesting involvement of cholinergic signaling and α7nAChRs. Overall, our findings support taVNS as a potential neuromodulatory treatment option for AD and warrant further mechanistic and clinical studies.

## 5. Conclusion

In a DNCB-induced mouse model of AD, 0.2 mA 15 Hz of taVNS reduced epidermal thickness, mast cells, ear swelling, and inflammatory markers (IL-4/IL-1β/TNF-α, MPO, p-NF-κB). α7nAChR blockade diminished these effects, implicating the cholinergic anti-inflammatory pathway. These data support taVNS as a feasible, noninvasive, non-pharmacological candidate therapy for AD.

## Ethics approval and consent to participate

All animal experiments were approved by the Institutional Animal Care and Use Committee of Korea University College of Medicine (Korea-2023-0098) and conducted in accordance with relevant guidelines and regulations.

## Data Availability

The data produced and/or evaluated in this study will be provided to the corresponding author upon a reasonable request.
